# Intestinal Perforation in ACTH-Dependent Cushing's Syndrome

**DOI:** 10.1155/2019/9721781

**Published:** 2019-03-13

**Authors:** Mariam Shahidi, Richard A. Phillips, Constance L. Chik

**Affiliations:** ^1^Division of Endocrinology and Metabolism, Department of Medicine, University of Alberta, Edmonton, Alberta, Canada T6G 2S2; ^2^Interior Health, Kelowna, BC, Canada and Division of Endocrinology, Department of Medicine, University of British Columbia, Canada

## Abstract

Previous studies have linked systemic glucocorticoid use with intestinal perforation. However, the association between intestinal perforation and endogenous hypercortisolism has not been well described, with only 14 previously published case reports. In this study, we investigated if intestinal perforation occurred more frequently in patients with ectopic ACTH syndrome and in those with a greater than 10-fold elevation of 24-hour urinary free cortisol level. Of 110 patients with ACTH-dependent Cushing's syndrome followed in two clinics in Canada, six cases with intestinal perforation were identified over 15 years. Age of patients ranged from 52 to 72, five females and one male, four with Cushing's disease and two with ectopic ACTH production, one from a pancreatic neuroendocrine tumor and one from medullary carcinoma of the thyroid. Five had diverticular perforation and one had intestinal perforation from a stercoral ulcer. All cases had their lower intestinal perforation when the cortisol production was high, and one patient had diverticular perforation 15 months prior to the diagnosis of Cushing's disease. As in previously reported cases, most had hypokalemia and abdominal pain with minimal or no peritoneal symptoms and this occurred during the active phase of Cushing's syndrome. Whereas all previously reported cases occurred in patients with 24-hour urinary free cortisol levels greater than 10-fold the upper limit of normal when measured and 11 of 14 patients had ectopic ACTH production, only one of our patients had this degree of hypercortisolism and four of our six patients had Cushing's disease. Similar to exogenous steroid use, patients with endogenous hypercortisolism also have a higher risk of intestinal, in particular diverticular, perforation and should be monitored closely for its occurrence with a low threshold for investigation and surgical intervention. Elective colonoscopy probably should be deferred until Cushing's syndrome is under control.

## 1. Introduction

The presence of colonic diverticular disease is widespread in the western world, with an estimated prevalence of 50% in those over age 60 and 65% in those over age 85 [1]. Approximately 15% of patients will develop complications, with diverticular perforation being a rare but serious one, given its high morbidity and mortality [2]. Although the specific mechanisms underlying such perforation remain unclear, proposed contributing factors include increased intracolonic pressure, impaired colonic mucosal barrier, altered colonic microflora, immunosuppression, and allergic predisposition [2–4]. Several studies have demonstrated an association between the use of corticosteroids, both systemic and inhaled, and intestinal perforation [2, 5–10].

To the best of our knowledge, there are only 14 published case reports of intestinal perforation, 12 with diverticular disease, in patients with Cushing's syndrome associated with elevated ACTH and cortisol levels [11–19], including 11 patients having ectopic ACTH syndrome. Moreover, all previously reported patients [11–19] had a greater than 10-fold elevation of 24-hour urinary free cortisol (UFC) level when measured. The purpose of our study was twofold: first to examine if intestinal perforation occurred more frequently in patients with ectopic ACTH syndrome and second if intestinal perforation only occurred in patients with a greater than 10-fold elevation of 24-hour UFC.

## 2. Methods 

In this study, we identified patients with ACTH-dependent Cushing's syndrome and intestinal perforation, in particular diverticular rupture, in one academic and one community-based endocrine practice in Canada. With a total catchment area of about 2 million people, both physicians care for the majority of patients with Cushing's syndrome. Over a period of 15 years, a chart audit was completed and a total of 110 patients with ACTH-dependent Cushing's syndrome were diagnosed, 95 with Cushing's disease and 15 with ectopic Cushing's syndrome. Of those, we identified six patients—two with ectopic ACTH production and four with ACTH-producing pituitary adenomas and intestinal perforation. The study was approved by the Research Ethics Board, University of Alberta, Edmonton, Alberta, and registered under ClinicalTrials.gov NCT03665064. Consent was obtained from the patients or their estates. Statistical analysis was performed using Student's t-test and analysis of variance.

## 3. Results

Of 110 patients with ACTH-dependent Cushing's syndrome, six cases with intestinal rupture were identified over 15 years, summarized in Table 1 and included as the last six cases of Table 2. Age of patients ranged from 52 to 72, with five females and one male; four had Cushing's disease and two had ectopic ACTH production, one from a pancreatic neuroendocrine tumor and one from medullary carcinoma of the thyroid (MCT). Patients had mild (patient 2) to florid (patients 1, 4, and 6) manifestations of Cushing's syndrome. Biochemical evaluation confirmed hypercortisolism, with higher levels of 24-hour UFC of 5296 nmol (patient 1) and/or plasma cortisol levels of 3243 nmol and 1925 nmol (patients 1 and 2, respectively) in patients with ectopic ACTH production. ACTH levels were 33.2 pmol/L and 49.9 pmol/L (patients 1 and 2, respectively). In comparison, 24-hour UFC and plasma cortisol levels were lower in patients with Cushing's disease, ranging from 348 to 1533 nmol (median 647.5 nmol) and 568 to 1228 nmol/L (median 961.5 nmol/L), respectively, with ACTH levels from 11 to 48 pmol/L (median 16.5 pmol/L). Five patients had diverticular perforation, and one had intestinal perforation from a stercoral ulcer. All cases had their lower intestinal perforation when the cortisol production was high, with patient 6 having a two-year history of symptoms of cortisol excess before her diverticular perforation 15 months prior to the diagnosis of Cushing's disease.

### 3.1. Case 1

**Figure 1:**
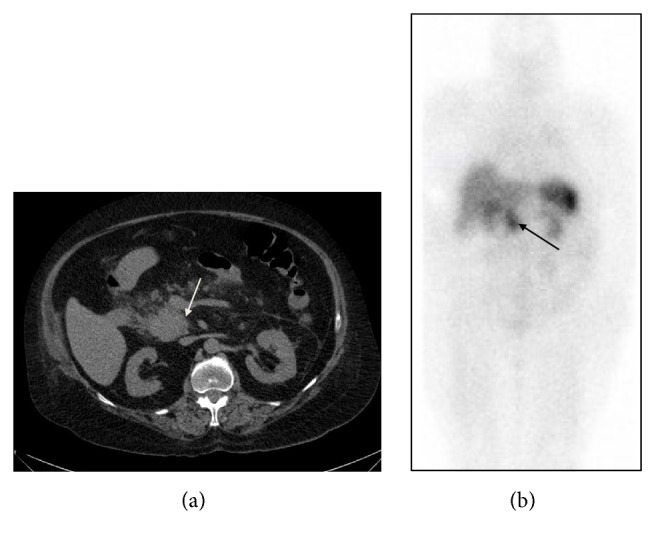
(a) CT abdomen of patient 1 showing a 6 cm pancreatic head/uncinate mass (indicated by an arrow). (b) Whole body image of octreotide scan, anterior view, demonstrating intense uptake of octreotide in the corresponding pancreatic head and uncinate process (indicated by an arrow), with no additional site of pathologic octreotide intake to suggest regional or distant metastases.

A 72-year-old woman presented to the emergency room with gastrointestinal symptoms and hypokalemia. Air in the sigmoid colon mesentery and a solid pancreatic head mass were noted on CT of the abdomen/pelvis (Figure 1(a)). Sigmoid diverticular perforation and a small pericolonic abscess were confirmed on subsequent Hartmann procedure. With cushingoid features, the Endocrinology service was consulted postoperatively. Collateral history from her family revealed a recent general decline in health and the development of signs and symptoms of hypercortisolism over the past 12 months. On exam, in addition to a cushingoid appearance, obesity (body mass index of 40) and mild hypertension (blood pressure 145/77 mmHg) were noted. Investigations were consistent with ectopic ACTH-dependent hypercortisolism from a pancreatic neuroendocrine tumor (Table 1). MRI sella was contraindicated because she had a pacemaker, but no pituitary adenoma was visible on CT of the head. Inferior petrosal sinus sampling was not completed because the patient was unstable. Treatment with metyrapone, followed later by the addition of ketoconazole was initiated with some benefit. Short-acting octreotide was added based on a positive octreotide scan (Figure 1(b)). Metyrapone and ketoconazole were discontinued several days later due to a significant drop in serum cortisol on octreotide. Given her recent Hartmann procedure and clinical instability, surgery was not an option. Almost one month after initiation of octreotide, she became hypotensive and unresponsive from a suspected intracranial hemorrhage. Her family decided against further medical intervention. A postmortem examination was not performed.

### 3.2. Case 2

A 56-year-old woman was referred to our center for management of pathologically confirmed multicentric MCT, postthyroidectomy. Genetic testing was negative for the ret-proto-oncogene mutation and there was no evidence of MEN 2A, with documented eucalcemia and negative 24-hour urinary metanephrines. Persistent postop elevation of serum calcitonin, MIBG, and octreotide scans suggested mediastinal disease which was confirmed upon mediastinal exploration. Over the next two years, calcitonin gradually rose and serial imaging showed metastatic foci of disease. 24-hour urine for metanephrines was negative and a 24-hour UFC was marginally elevated at 320 nmol (normal < 300 nmol), with a repeat measurement being normal at 208 nmol. She had no overt cushingoid features on physical exam. Subsequent exploratory laparoscopic surgery revealed liver metastases, making further surgery unsuitable. The decision was made to treat medically with octreotide.

At age 61, five years after her initial diagnosis with MCT, her condition deteriorated. She was noted to have mild cushingoid features and further investigations revealed likely ectopic Cushing's syndrome from her MCT (Table 1). She was admitted to hospital with complaints of dyspnea, mild abdominal pain, and profound hypokalemia of 2.4 mmol/L and required intubation and ventilation for progressive respiratory distress. On CT scan, there was free intraperitoneal air and an abnormal loop of bowel in the region of the sigmoid colon, consistent with perforated sigmoid diverticular disease. She opted for palliative care and was deceased two weeks later.

### 3.3. Case 3

A 58-year-old woman was referred for evaluation of Cushing's syndrome following a one-year history of skin thinning, easy bruising, hirsutism, worsening headaches, a 25 pound weight gain, and a new diagnosis of type 2 diabetes mellitus. Physical examination revealed facial plethora, supraclavicular and dorsocervical fat pads, and mild proximal myopathy. Subsequent laboratory and imaging investigations were consistent with Cushing's disease (Table 1). Transsphenoidal surgery was completed and pathology revealed a corticotroph adenoma. Steroid replacement, which was started post-op, was tapered over 18 months with resolution of cushingoid features and no biochemical evidence of disease recurrence.

**Figure 2:**
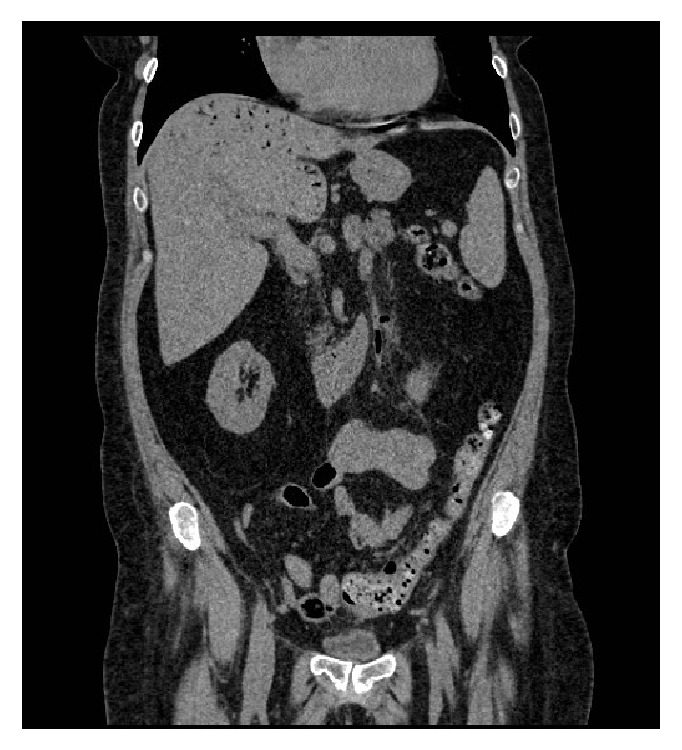
CT abdomen/pelvis of patient 3. Representative CT showing numerous tubular structures within the liver filled with gas extending to the subcapsular liver surface, felt to be secondary to a perforated diverticulum in the sigmoid colon and secondary abscess.

However, at age 68, she had recurrent signs and symptoms of Cushing's disease which was confirmed on biochemical testing (Table 1). She was started on pasireotide, on which she developed liver enzyme elevation and worsening glycemic control. She also experienced left lower quadrant abdominal pain with ongoing constipation. Pasireotide was discontinued and abdominal ultrasound revealed an extensive gas collection within the intrahepatic portal branches and main portal vein. CT abdomen/pelvis showed a small pelvic abscess adjacent to the sigmoid colon, representing a secondary abscess from a perforated diverticulum (Figure 2). She responded to intravenous antibiotics. Three months later, she was readmitted to hospital with a colocutaneous fistula, which resolved with conservative management alone.

**Figure 3:**
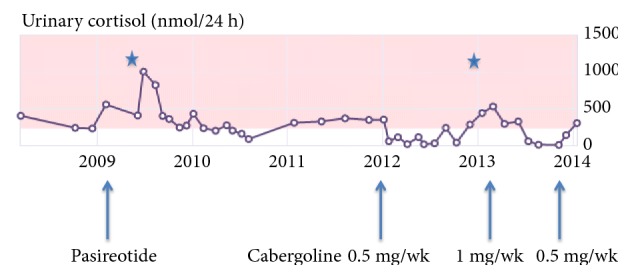
24-hour urinary free cortisol levels from 2008 to 2014 of patient 3. Episodes of perforation indicated by *∗*, the first episode occurred while on pasireotide and the second one during colonoscopy.

Off all treatment for more than two years, she had ongoing cyclical Cushing's disease and a residual microadenoma on MRI sella that was not amenable to surgery given its proximity to the optic chiasm. Cabergoline was initiated and her 24-hour UFC levels normalized for seven months, following which levels were intermittently elevated (Figure 3). At the age of 71, she sustained a diverticular tear during screening colonoscopy when her 24-hour UFC was above normal. Emergent laparotomy was performed, during which she had a right hemicolectomy.

### 3.4. Case 4

A 71-year-old woman, unwell for three months with recurrent exacerbations of chronic obstructive pulmonary disease and congestive heart failure, was hospitalized multiple times at her local hospital. Her internist suspected Cushing's syndrome based on the presence of suggestive signs and symptoms. Biochemical and dynamic testing supported this diagnosis (Table 1). During this time, she was experiencing intermittent lower abdominal pain. With recurrent abdominal pain one month later, CT abdomen/pelvis showed chronic perforation of a hollow viscus on a background of extensive sigmoid diverticular disease, confirmed on subsequent barium enema. With adequate response to antibiotic treatment, no surgical intervention was required. A repeat 24-hour UFC was normal, thus supporting the presence of cyclical Cushing's syndrome.

One month later, with a recurrence of cushingoid features, transsphenoidal hypophysectomy was performed. Bisection of the pituitary gland yielded a gush of tumor fluid but no tissue was available for immunostaining. She remained eucortisolemic nine months after surgery, and a repeat CT abdomen showed an enterocolic fistula within the left hemipelvis, but no peridiverticular abscess or perforation.

### 3.5. Case 5

A 51-year old male was suspected of having Cushing's syndrome based on his presentation with purpura, central obesity, facial plethora, and hypertension. Subsequent investigations confirmed ACTH-dependent Cushing's syndrome, likely of pituitary etiology (Table 1). During his first transsphenoidal surgery, tumor was visualized but no sample was obtained for pathological analysis. With persistent postop hypercortisolism, repeat transsphenoidal surgery was completed two months later, with a corticotroph adenoma being confirmed on pathology. The patient remained hypercortisolemic after the second surgery and one year later, cabergoline 1 mg twice weekly was used for persistent and moderately elevated 24-hour UFC levels. At age 54, during a period of medication nonadherence, 24-hour UFC increased to twice normal (Table 1). Two months later, he presented to hospital with a three-day history of worsening left lower quadrant abdominal pain. CT abdomen showed a large collection of stool and gas centrally in the mesentery, on the background of multiple diverticulae. Surgery revealed a 1.5 × 3 cm perforation of the sigmoid colon, with features more consistent with a perforated stercoral ulcer, rather than a diverticular perforation. Distal sigmoidectomy with creation of an end colostomy was performed. The patient did well and remained eucortisolemic on cabergoline 1 mg twice weekly.

### 3.6. Case 6

A 53-year-old woman was referred for investigation of hypercortisolism following a two-year history of easy bruising, proximal muscle weakness, and poor wound healing after surgery for diverticulitis and a vaginal fistula 15 months earlier. Pathology showed perforated diverticulitis of the rectosigmoid colon, and her surgery was complicated by deep vein thrombosis and pulmonary embolism. Additional history revealed insomnia, depression, obesity, and hypertension. Mild diverticular disease had been diagnosed three years earlier when she underwent investigations for left lower quadrant pain. Physical examination revealed classic features of cortisol excess with facial plethora, supraclavicular and dorsocervical fat pads, and mild proximal myopathy. Investigations, including inferior petrosal sinus sampling, were consistent with Cushing's syndrome of a pituitary etiology (Table 1). The patient underwent transsphenoidal resection and pathology confirmed a corticotroph adenoma.

## 4. Discussion

Our study of six patients and the previously published reports (summarized in Table 2) highlight the association between endogenous hypercortisolemia from ACTH-dependent Cushing's syndrome and intestinal perforation. A notable difference between our six patients and previous reports is that four of six patients (66.7%) had Cushing's disease whereas 11 of 14 previously described patients had ectopic ACTH syndrome, and only three patients (21.4%) had Cushing's disease. The greater proportion of Cushing's disease patients in our series most likely accounts for the female predominance (five of six patients) and the modest level of hypercortisolemia (3.6-fold increase). Only one of our patients had a greater than 10-fold elevation of 24-hour UFC level. The 14 previously reported patients comprised seven females and all had a greater than 10-fold elevation of 24-hour UFC when measured. The average age when intestinal perforation occurred was 63 years in our series, which is similar to the previously described cases, where the average age was 60 years. We found the average interval between onset of Cushing's symptoms and intestinal rupture to be 9.8 months, similar to previous reports with an average interval of 9.9 months. In both our series and previous cases, the intestinal perforation occurred only when the patients were actively hypercortisolemic and not during remission; however intestinal rupture may not occur when cortisol production is highest as was observed in patient 3, possibly reflecting another contributing factor such as the duration of elevated cortisol production.

Accepted general risk factors for diverticular perforation include aging, collagen vascular disease, altered histamine metabolism, immunosuppression, and corticosteroid use [3]. In our series, six of 110 (5.5%) patients with Cushing's syndrome had lower intestinal rupture. We included a case of stercoral ulcer perforation with background diverticular disease as the mechanisms would appear similar. A recent meta-analysis based on pooled data from five case control studies found increased odds of diverticular perforation in steroid users (odds ratio 9.08; range 2.17-31.9) [20]. Based on limited studies, the estimated incidence of diverticular rupture with steroid use varies between 0.7% and 2.7%, with the higher rate reflecting high dose and longer duration of steroid use [6, 8]. As patients with ectopic ACTH production commonly have gross elevation of cortisol levels and those with Cushing's disease potentially a long duration of elevated cortisol level, this may account for the higher incidence of intestinal rupture in our cohort of patients. However, unlike the other series of six patients reported by Sater et al. [19], our six patients had much lower 24-hour UFC levels, including four with Cushing's disease, and could reflect a difference in the referral pattern.

In large case series, overall mortality from perforated diverticular disease ranges between 12% and 36% [2]. However, mortality rates in those with concurrent exogenous glucocorticoid use ranges from 27% to 100% [5, 21, 22]. In our case series, patients with Cushing's syndrome experienced intestinal perforation, either at or around the time of initial investigation of Cushing's syndrome, as in our first patient and several previous reports [11–15, 18], or as a later clinical finding following known hypercortisolism (patients 2 to 5), or months prior to the formal diagnosis of Cushing's syndrome (patient 6). Two of our patients did not survive the event. Our third patient was treated conservatively following abscess and fistula formation from a spontaneous diverticular perforation, only to ultimately require partial colectomy for management of an iatrogenic diverticular tear during colonoscopy.

Diverticular perforation resulting from hypercortisolism may be due to several mechanisms, such as compromised colonic wall integrity from decreased collagen turnover, and disruption of mucosal-protective prostacyclin synthesis [3, 4]. The immunosuppressive effects of hypercortisolism, whether endogenous or exogenous, may contribute to initial lack of containment of a perforation and impaired healing [9]. Furthermore, cortisol excess may mask classic symptoms of perforation, leading to a delay in diagnosis and treatment and a probable increase in morbidity and mortality [9] as emphasized in the recent case series [19]. Older studies have proposed that masking of symptoms occurs due to a reduction in the availability and concentration of immunoreactive cells and substances to areas of injury or inflammation, as well as from an impairment of interactions required for cellular immune defense [2, 3, 23]. In steroid-treated patients, this may result in a diagnostic delay of 8 to 14 days [6, 23], and one may expect a similar lag time in those with endogenous hypercortisolism. Indeed, most of our patients only had mild abdominal pain and few peritoneal symptoms at the diagnosis of intestinal rupture. Only one patient with Cushing's syndrome secondary to a pheochromocytoma, reported by Flynn et al. [18], had severe abdominal pain with peritonism from perforation at the splenic flexure at the time she underwent investigation for cortisol excess.

Only recently has the glucocorticoid-induced TNF-alpha receptor (GITR) been implicated in the pathogenesis of steroid-induced complicated diverticular disease [3, 24–27]. GITR, a member of the TNF receptor superfamily, modulates immune function and inflammatory response [27]. It is overexpressed in the inflammatory infiltrate of patients with complicated diverticulitis [25]. In addition to being induced by proinflammatory states, activation of GITR appears to promote such a state by stimulating most immune cell types, as well as downregulating suppressor T-lymphocytes [25, 27]. GITR signaling also increases expression of the enzyme involved in remodeling of the colon wall, matrix metalloproteinase-9, another mechanism which may promote perforation [25]. Of interest, the use of anti-GITR antibodies as possible anti-inflammatory drugs has been studied but is limited by their structural complexity and concerns about the development of autoimmunity [25, 27]. In complicated diverticulitis, exogenous steroids increase recruitment of inflammatory cells, such as macrophages and neutrophils, and delay neutrophil apoptosis [24]. It is probable that endogenous hypercortisolism has similar effects on immune function in the setting of complicated diverticulitis.

Other than corticosteroids, several studies have demonstrated a positive, although weaker, association between nonsteroidal anti-inflammatory drugs (NSAIDs) and opioids and diverticular perforation [2, 9, 28, 29]. Deficiency of prostaglandins and suppressed secretion of protective substances, like bicarbonate and mucin, by NSAIDs can lead to damage of surface epithelial cells and increased permeability of the colonic mucosa [9]. Opioids may slow colonic transit time and raise intraluminal pressure [28]. With increased recognition of patients with Cushing's syndrome and colonic intestinal or diverticular perforation, use of medications potentially contributing to intestinal perforation should be minimized. Reduction of modifiable risk factors, like smoking, alcohol use, fiber deficiency, and excess red meat, is also recommended [2]. Other pharmacologic agents, such as calcium channel blockers and statins, have been linked to a reduction in perforation risk [28, 29]. Calcium channel blockers may reduce intracolonic pressure by decreasing the strength and duration of colonic contractions, while statins have anti-inflammatory properties and were associated with a 50% risk reduction in one study [28, 29]. Choice of these agents when treatment is indicated for hypertension and dyslipidemia, and common metabolic consequences of hypercortisolism could be considered, and their routine use in these patients merits future investigation.

Interestingly, a history of steroid use may also be a risk factor for diverticular perforation, increasing risk by as much as 70%, compared with over 300% in current steroid users [29]. Therefore, patients with a history of Cushing's syndrome may also have a life-long increased risk of perforated diverticular disease, even with curative treatment. However, in the 20 reported cases to date, intestinal perforation was observed only during the active phase of Cushing's syndrome.

## 5. Conclusion

In contrast to previous reports, only one of our six patients with intestinal perforation had 24-hour UFC level greater than 10-fold the upper limit of normal, and only two patients had ectopic ACTH production. As with chronic exogenous corticosteroid use, our additional cases further support intestinal/diverticular rupture being an infrequent but potentially fatal complication of diverticular disease in patients with Cushing's syndrome. Physicians should promptly investigate patients with hypercortisolism and apparently minor gastrointestinal symptoms or suspected diverticular disease, as early detection and management of intestinal/diverticular perforation probably can help reduce mortality. Given the risk of perforation, management of hypercortisolism prior to elective colonoscopy is a consideration.

## Figures and Tables

**Table 1:** Features of Cushing's syndrome of our six patients with intestinal perforation.

Case	Age Gender	Presentation	Laboratory Investigations	Imaging Investigations	Etiology	Treatment
1	72, Female	Nausea, vomiting, abdominal pain, hypokalemia from diverticular rupture; cushingoid features, hypertension and type 2 diabetes mellitus	*At presentation/around time of perforation:* Cortisol 3243 nmol/L, ACTH 33.2 pmol/L UFC 5296 nmol Chromogranin A – 81 U/L Glucagon – 91.2 ng/L	CT head – negative CT abdomen - 6 cm pancreatic mass with calcifications Octreotide scan – uptake within pancreatic mass	Biopsy confirmed pancreatic neuroendocrine tumor	Hartmann procedure; ketoconazole, metyrapone, and octreotide for hypercortisolism

2	61, Female	Known metastatic medullary thyroid carcinoma, hypokalemia, dyspnea, abdominal pain from diverticular rupture	*Around time of perforation:* Cortisol 1925 nmol/L, ACTH 49.9 pmol/L UFC – not done	CT abdomen – 2 cm bilateral adrenal masses with no uptake on MIBG scan	Presumed ectopic from medullary thyroid carcinoma	Conservative management

3	68, Female	Known Cushing's disease with recurrence ten years after surgery; worsening glycemic control, left lower quadrant abdominal pain, elevated liver enzymes from diverticular rupture	*At presentation:* Cortisol 600 nmol/L, ACTH 16.3 pmol/L UFC 1088 nmol (<300 nmol) *Around time of perforation:* Cortisol 797 nmol/L, ACTH 48.0 pmol/L UFC 410 nmol *Before second rupture:* Cortisol 568 nmol/L, ACTH 18.7 pmol/L UFC 348 nmol	*At presentation:* MRI sella – left-sided adenoma *At time of recurrence:* MRI sella - 3 mm lesion not amenable to surgery	Cushing's disease recurrence, corticotroph adenoma	IV antibiotics, cabergoline after initial rupture; Right hemicolectomy for diverticular tear

4	71, Female	Hypertension, type 2 diabetes mellitus, hypokalemia, lower abdominal pain; chronic perforation, extensive sigmoid diverticular disease	*At presentation/around time of perforation:* Cortisol 1228 nmol/L, ACTH 13.5 pmol/L UFC 1533 nmol (N <220 nmol)	MRI sella - 3 mm adenoma; CT abdomen – stable 2 cm bilobed left adrenal adenoma/ hyperplasia	Cushing's disease, corticotroph adenoma	IV antibiotics before transsphenoidal surgery

5	54, Male	Persistent Cushing's disease post-surgery, on cabergoline; lower abdominal pain, sigmoid colon perforation	*At presentation:* Cortisol 469 nmol/L after 1 mg dexamethasone, ACTH 19.4 pmol/L, UFC 374 nmol (N<220 nmol) *Around time of perforation:* UFC 328 nmol (N<166 nmol)	MRI sella – normal	Cushing's disease, corticotroph adenoma	Surgical management of stercoral ulcer perforation and resumption of cabergoline

6	52, Female	Abdominal pain with symptoms of vaginal fistula and symptoms of of hypercortisolism	*15 months post-surgical repair of diverticular rupture:* Cortisol 1125 nmol/L, ACTH 11 pmol/L UFC 885 nmol	MRI sella – 4 mm left-sided adenoma	Cushing's disease, corticotroph adenoma	Surgical management

Reference range for plasma cortisol (85-620 nmol/L), ACTH (2.2-10.1 pmol/L), and UFC (<230 nmol) if not specified.

**Table 2:** Previous and current cases of patients with Cushing's syndrome and intestinal perforation.

Reference	Age	Gender	Highest UFC (nmol/24 h) / Plasma cortisol (PC, nmol/L)	Cause of Cushing's syndrome	Hypokalemia	^*∗*^Time from onset of CS (months)	Required Sx and/or surgical finding
Drake et al. [11]	35	Male	PC = 1442	Islet cell tumor	Yes	1	Duodenal perforation and rupture of pancreatic pseudocyst

Lutgers et al. [12]	55	Female	10152	Right pheochromocytoma	Yes	1	Perforated sigmoid diverticulum

De Havenon et al. [13]	71	Female	PC = 2593 after 8 mg dexamethasone	Cushing's disease	Yes	9	Diverticular rupture

Hara et al. [14]	79	Male	PC = 1230	Cushing's disease	Yes	6	Perforation of colonic diverticulum

Kaya et al. [15]	70	Male	PC = 1432	Small cell lung carcinoma	Yes	1	Diverticular rupture in the rectosigmoid area

Matheny et al. [16]	67	Male	11119	Metastatic medullary carcinoma of thyroid	Yes	4	Drainage and IV antibiotics for perforated diverticulum

Dacruz et al. [17]	60	Female	4481	Metastatic parotid tumor	Yes	5	Perforated sigmoid diverticulitis

Flynn et al. [18]	63	Female	12465	Pheochromocytoma	Yes	1	Hemicolectomy for perforation at the splenic flexure

Sater et al. [19]	80	Female	5601	Lung carcinoid	Yes	36	Acute diverticulitis perforation and serositis

Sater et al. [19]	60	Female	72726	Metastatic islet cell carcinoma	Yes	36	Sigmoid resection with colostomy

Sater et al. [19]	31	Male	1297	Cushing's disease	No	20	Acute and chronic inflammation of left colon and retroperitoneal tissue with focal abscess

Sater et al. [19]	52	Female	2371	Lung carcinoid	Yes	4	Acute diverticulitis with rupture and abscess formation

Sater et al. [19]	67	Male	3836	Ectopic ACTH (not localized)	No	10	Acute sigmoid diverticular rupture rupture complicated by a peri-colic abscess

Sater et al. [19]	51	Male	13552	Metastatic thymic carcinoma	Yes	4	Partial colon resection with colostomy

Current	72	Female	5296	Pancreatic neuroendocrine tumor	Yes	12	Hartmann procedure for diverticular rupture

Current	61	Female	PC = 1925	Metastatic medullary carcinoma of thyroid	Yes	12	Palliative

Current	68	Female	410	Cushing's disease	No	12	IV antibiotics (first episode) Right hemicolectomy (second episode)

Current	71	Female	1533	Cushing's disease	Yes	4	IV antibiotics

Current	54	Male	374	Cushing's disease	No	3	Sigmoid colon perforation

Current	52	Female	885	Cushing's disease	Yes	16	Colectomy for diverticular rupture

*∗*An estimate only as some patients had intestinal rupture after definitive treatment of their Cushing's syndrome (CS).

## Data Availability

The data used to support the findings of this study are available from the corresponding author upon request.
